# Autism in a Child With X-linked Agammaglobulinemia

**DOI:** 10.7759/cureus.21951

**Published:** 2022-02-06

**Authors:** Adam Bied, Susan Njuguna, Ritvij Satodiya

**Affiliations:** 1 Psychiatry, Stony Brook University, New York, USA; 2 Psychiatry, ABC Medical, Reading, USA; 3 Child and Adolescent Psychiatry, New York University Grossman School of Medicine, New York, USA

**Keywords:** antibody deficiency syndrome, pervasive developmental disorder, immunodeficiency, autism, agammaglobulinemia

## Abstract

A growing evidence base has implicated immune dysfunction in the etiology of some cases of autism spectrum disorder. The precise relationship between immune disorders and autism spectrum disorder remains unclear. Herein we report a 14-year-old-male with agammaglobulinemia, who was diagnosed with autism spectrum disorder, and who has received exogenous immunoglobulins regularly for most of his life. This case study supports current theories implicating antibody deficiencies in some individuals with an autism spectrum disorder. Our case will add to the growing literature of understanding the connection between immune deficiencies in the pathogenesis of autism.

## Introduction

Autism spectrum disorder is a complex neurodevelopmental syndrome with growing evidence involving genetics and immune system dysfunction [1-3]. Immune system dysregulation, autoimmunity and reduced serum levels of immunoglobulins have all been associated with some cases of autism spectrum disorder [4]. Conversely, administration of intravenous immunoglobulin has been shown to improve the core symptoms among some children experiencing the disorder [5,6]. The efforts to understand the role of the immune system in autism and using this knowledge for development of therapeutics has been ongoing. X-linked agammaglobulinemia is a rare primary immunodeficiency disorder characterized by a near total lack of antibody production, attenuated or absent B lymphocyte and plasma cell activity, and a depravity or absence of lymphocytes expressing B-cell marker, cluster of differentiation 20 (CD20), and the cluster of differentiation 19 (CD19) [7]. Herein, we report an X-linked agammaglobulinemia affected individual with a comorbid autism spectrum disorder.

## Case presentation

A 14-year-old male with X-linked agammaglobulinemia from non-consanguineous parents presented to the lead author’s hospital for behavioral problems. The pregnancy was reported to be full term though significant for first-trimester vaginal bleeding and periodic emesis. He was described as a “fussy” and “very active baby” by his mother who also reported significant delays and subsequent impairment in language. He later began headbanging, displayed poor eye contact, and was sensitive to both light and sound. He was also reported to display a peculiar tendency to spin objects. He was diagnosed with autism spectrum disorder, per Diagnostic and Statistical Manual of Mental Disorders, Fourth Edition (DSM-IV) criteria, by age two and underwent various psychosocial and psychopharmacological treatments thereafter to address comorbid irritability and behavioral problems. A review of his records at the author’s institution revealed multiple emergency psychiatric presentations and several acute inpatient hospitalizations for behavioral issues and mood problems. Although he was verbal during his evaluation with the lead author, he had a deficiency in pragmatics and an obsessive interest in dinosaurs. At the time of his assessment, he was housed in a residential treatment facility. He also had a history of recurrent bacterial infections commencing around six months of age and was initially managed with antimicrobials alone. Subsequent evaluations revealed a total and sustained absence of B-lymphocytes and a sustained depressed level of serum immunoglobulins. The child’s hematologist subsequently diagnosed him with X-linked agammaglobulinemia and initiated him on monthly infusions of immunoglobulins. His treatment regimen at the time of his evaluation was 10% concentration dosed 300 mg per kilogram administered monthly. No family history of psychiatric illness was noted in any of the child’s immediate family members.

## Discussion

To the knowledge of the authors, this is one of the first reported cases of a child with X-linked agammaglobulinemia and an autism spectrum disorder. Minimal literature thus far exists regarding the relationship between X-linked agammaglobulinemia and autism. Prior studies have estimated X-linked agammaglobulinemia occurs with a prevalence of between two and eight per one million and it is considered to be preserved in the population by the occurrence of new mutations. Most individuals with the X-linked agammaglobulinemia therefore have no family history of the disorder and are the first manifestation in their family of a new mutation [8]. Autism spectrum disorder, alternatively, has a substantial hereditary basis [9]. Studies of twins reveal heritability as high as 0.9 for autism spectrum disorder, and siblings of those with autism are approximately at twenty-five times more risk than the general population [10]. If X-linked agammaglobulinemia is a putative factor in such cases of autism spectrum disorder, one may therefore expect minimal family history of the disorder and no greater risk of the autism diagnosis than immunocompetent family members.

While there exists no literature regarding autism in X-linked agammaglobulinemia, there have been several models implicating primary immunodeficiency with an autism spectrum disorder. Some authors have proposed a genetic cause shared between both primary immunodeficiency disease and an autism spectrum disorder. Favoring this several primary immunodeficiency diseases, including hyper IgE syndrome, common variable immunodeficiency, and IgA deficiency have been shown to be risk factors for autism spectrum disorder [6,10,11]. Prior research attributed this to a shared genetic mechanism proposing that the proximal portion of chromosome 4q contained genes responsible for both immunoglobulin production and some cases of autism spectrum disorder [6,9]. The primary immunodeficiency-autism haplotype is theorized to result in an autism spectrum disorder phenotype comorbid with one or more primary immunodeficiencies. Other authors have implicated immune dysfunction with the pathogenesis of autism spectrum disorder. Defects in lymphocyte activity, both B and T lineage, along with depressed serum immunoglobulins have been shown to occur among some children with autism [11]. Plasma IgG and IgM have been shown to be reduced among some individuals with the autism spectrum disorder diagnosis and immunoglobulin levels have been shown to inversely predict Aberrant Behavior Checklist scores for both autistic and normally developing children alike [3]. An alternative model proffered by some authors has implicated excessive immune activity as a mechanism. Favoring this the primary immunodeficiency diseases like hyper IgE syndrome, common variable immunodeficiency, and IgA deficiency have also been shown to be independent risk factors for autoimmunity [12]. Autoimmunity has also been observed in higher prevalence among immunocompetent individuals with autism than in the general population [13]. Adults with autism have been shown to have higher proportions of B-lymphocytes when compared to healthy controls [14]. B-lymphocytes specific to certain infections, are also found to be more common among those with autism spectrum disorder, are predictive of the diagnosis, and have been correlated to the severity of repetitive behaviors in autism spectrum disorder [15]. Consistent with this, B-lymphocyte cytokines are elevated in individuals with autism spectrum disorder regardless of immune status [16]. 

Our case diverges from the other primary immunodeficiency diseases in several respects. X-linked agammaglobulinemia is X-linked and hence far removed from the genetic regions implicated in the other primary immunodeficiency diseases. Once more, X-linked agammaglobulinemia unlike other primary immunodeficiency diseases appears to confer no greater risk of autoimmunity [17]. While some individuals with autism spectrum disorder appear to have more pronounced B-lymphocyte activity, our case has essentially none. At a minimum reconciling our case with that of the broader autism spectrum disorder and primary immunodeficiency literature is challenging. Further research is therefore encouraged particularly with regard to the psychiatric comorbidity in individuals with X-linked agammaglobulinemia. Barring such work, clinicians may wish to consider employing psychiatric assessments more readily when faced with established cases of X-linked agammaglobulinemia, may wish to assess the impact of immunoglobulin treatments on autism spectrum disorder symptoms and may wish to solicit more detailed family history, particularly for psychiatric ailments.

## Conclusions

Our case is notable for profound impairment in immunoglobulin production in combination with an autism spectrum disorder. Like the other primary immunodeficiencies implicated in autism spectrum disorder, this one is notable for pronounced immunoglobulin deficiency. Furthermore, the candidate genes postulated in this syndrome are found on the X chromosome rather than chromosome 4. This case, if generalizable, would suggest that a different mechanism, perhaps directly related to immunoglobulin activity is related to the pathogenesis of autism. Further research regarding this immunodeficiency syndrome and its relationship with autism spectrum disorder is encouraged.
